# Flood‐irrigated agriculture mediates climate‐induced wetland scarcity for summering sandhill cranes in western North America

**DOI:** 10.1002/ece3.10998

**Published:** 2024-03-05

**Authors:** J. Patrick Donnelly, Daniel P. Collins, Jeffrey M. Knetter, James H. Gammonley, Matthew A. Boggie, Blake A. Grisham, M. Cathy Nowak, David E. Naugle

**Affiliations:** ^1^ Intermountain West Joint Venture—U.S. Fish and Wildlife Service Migratory Bird Program Missoula Montana USA; ^2^ W.A. Franke College of Forestry and Conservation University of Montana Missoula Montana USA; ^3^ Idaho Department of Fish and Game Boise Idaho USA; ^4^ Colorado Parks and Wildlife Fort Collins Colorado USA; ^5^ Department of Natural Resources Management Texas Tech University Lubbock Texas USA; ^6^ Oregon Department of Fish and Wildlife Ladd Marsh Wildlife Area La Grande Oregon USA; ^7^ U.S. Fish and Wildlife Service Southwest Region Migratory Bird Program Albuquerque New Mexico USA

**Keywords:** flood‐irrigated agriculture, greater sandhill crane, private lands, random Forest, species distribution model, wetlands

## Abstract

Information about species distributions is lacking in many regions of the world, forcing resource managers to answer complex ecological questions with incomplete data. Information gaps are compounded by climate change, driving ecological bottlenecks that can act as new demographic constraints on fauna. Here, we construct greater sandhill crane (*Antigone canadensis tabida*) summering range in western North America using movement data from 120 GPS‐tagged individuals to determine how landscape composition shaped their distributions. Landscape variables developed from remotely sensed data were combined with bird locations to model distribution probabilities. Additionally, land‐use and ownership were summarized within summer range as a measure of general bird use. Wetland variables identified as important predictors of bird distributions were evaluated in a post hoc analysis to measure long‐term (1984–2022) effects of climate‐driven surface water drying. Wetlands and associated agricultural practices accounted for 1.2% of summer range but were key predictors of occurrence. Bird distributions were structured by riparian floodplains that concentrated wetlands, and flood‐irrigated agriculture in otherwise arid and semi‐arid landscapes. Findings highlighted the role of private lands in greater sandhill crane ecology as they accounted for 78% of predicted distributions. Wetland drying observed in portions of the range from 1984 to 2022 represented an emerging ecological bottleneck that could limit future greater sandhill crane summer range. Study outcomes provide novel insight into the significance of ecosystem services provided by flood‐irrigated agriculture that supported nearly 60% of wetland resources used by birds. Findings suggest greater sandhill cranes function as a surrogate species for agroecology and climate change adaptation strategies seeking to reduce agricultural water use through improved efficiency while also maintaining distinct flood‐irrigation practices supporting greater sandhill cranes and other wetland‐dependent wildlife. We make our wetland and sandhill crane summering distributions available as interactive web‐based mapping tools to inform conservation design.

## Graphical abstract

Summer range for greater sandhill cranes (Antigone canadensis tabida) was reconstructed in western North America using movement data from 120 GPS‐tagged individuals to determine how landscape composition shaped their distributions. Wetlands and associated agricultural practices accounted for 1.2% of their range but were key predictors of occurrence. Bird distributions were patterned primarily by riparian floodplains that concentrated wetlands, and flood‐irrigated agriculture in otherwise arid and semi‐arid landscapes. Findings suggest greater sandhill cranes function as an umbrella species for agroecology and climate change adaptation strategies seeking to reduce agricultural water use through improved efficiency while also maintaining distinct flood‐irrigation practices supporting greater sandhill cranes and other wetland‐dependent wildlife.

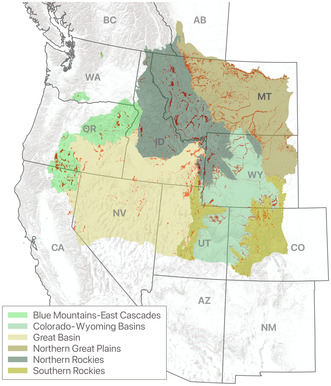

## 1 INTRODUCTION

Knowing a species's geographical extent is often the first step in understanding its ecology and is critical to informing conservation planning (Guisan & Tingley, 2013). In many regions of the world, even basic information about species distribution is lacking (Pimm & Jenkins, 2014), forcing natural resource managers to answer complex ecological questions with incomplete data. Increased frequency and severity of extreme weather and climate events are triggering resource bottlenecks that can act as powerful demographic constraints on fauna and exacerbate other human‐induced pressures such as land‐use change (Maron et al., 2015). Addressing these evolving information gaps requires the integration of GPS animal tracking technologies with satellite imagery and cloud computing to efficiently monitor species' response to changing ecosystem conditions at regional and continental scales (Pimm & Alibhai, 2015). Such information can provide the foundation for science‐based management necessary for assessing emerging biodiversity risks (Wiens & Stralberg, 2009).

In western North America, greater sandhill cranes (*Antigone canadensis tabida*, hereafter sandhill cranes) are iconic migratory waterbirds representative of wetland and riparian (hereafter ‘wetland’, Armbruster, 1987) ecosystems. Sandhill crane's annual life cycle (i.e., wintering, migration, and summering) is closely linked to water availability and hydrologic cycles that drive wetland function and irrigated agriculture (Donnelly & King, 2021). During summering periods, for example, birds defend traditional breeding territories around wetlands to reduce predator risks by building nest mounds in standing water (Austin et al., 2007). Emergent vegetation at these sites, in turn, supports important food resources for sandhill crane colts, particularly during late summer when seasonal drought limits foraging opportunities in desiccated uplands (Drewien & Bizeau, 1974). Increasing water scarcity driven by warming temperatures and prolonged droughts raises concerns over climate resilience in wetland ecosystems supporting sandhill crane populations. Additionally, reliance on privately owned agricultural lands could expose summering birds to an increased risk of land‐use change driven by climate adaptation strategies that shift cropping and water usage to practices incompatible with bird needs (Austin et al., 2018).

Characterizing sandhill crane summering distributions and their relationship to rural private lands agriculture fills a crucial gap in western North America's climate change adaptation strategies. Recent studies associate broad‐scale ecosystem services with riparian floodplains supporting flood‐irrigated agriculture that bolster climate resilience through groundwater recharge sustaining instream flows (Gordon & Paige, 2020; Kendy & Bredehoeft, 2006) and coldwater fisheries (Blevins et al., 2016). In some regions, these agroecosystems complement broader wetland function, particularly flood‐irrigation tied to grass‐hay production that supports nearly 60% of temporary wetlands in the Intermountain West, USA (Donnelly et al., 2023), providing vital resources for migratory waterbirds (Moulton & Carlisle, 2022). Well‐intended efforts to curtail climate change impacts through government programs that increase agricultural water use efficiencies by targeting perceived wasteful flood‐irrigation could unintentionally decouple long‐standing land‐use practices benefiting sandhill cranes and other wetland‐dependent wildlife. Documenting sandhill crane summering distributions will provide an important addendum to climate adaptation strategies seeking to reduce overall water consumption on public and private lands through an improved understanding of tradeoffs between changing water management and ecosystem services benefiting these birds.

**FIGURE 1:**
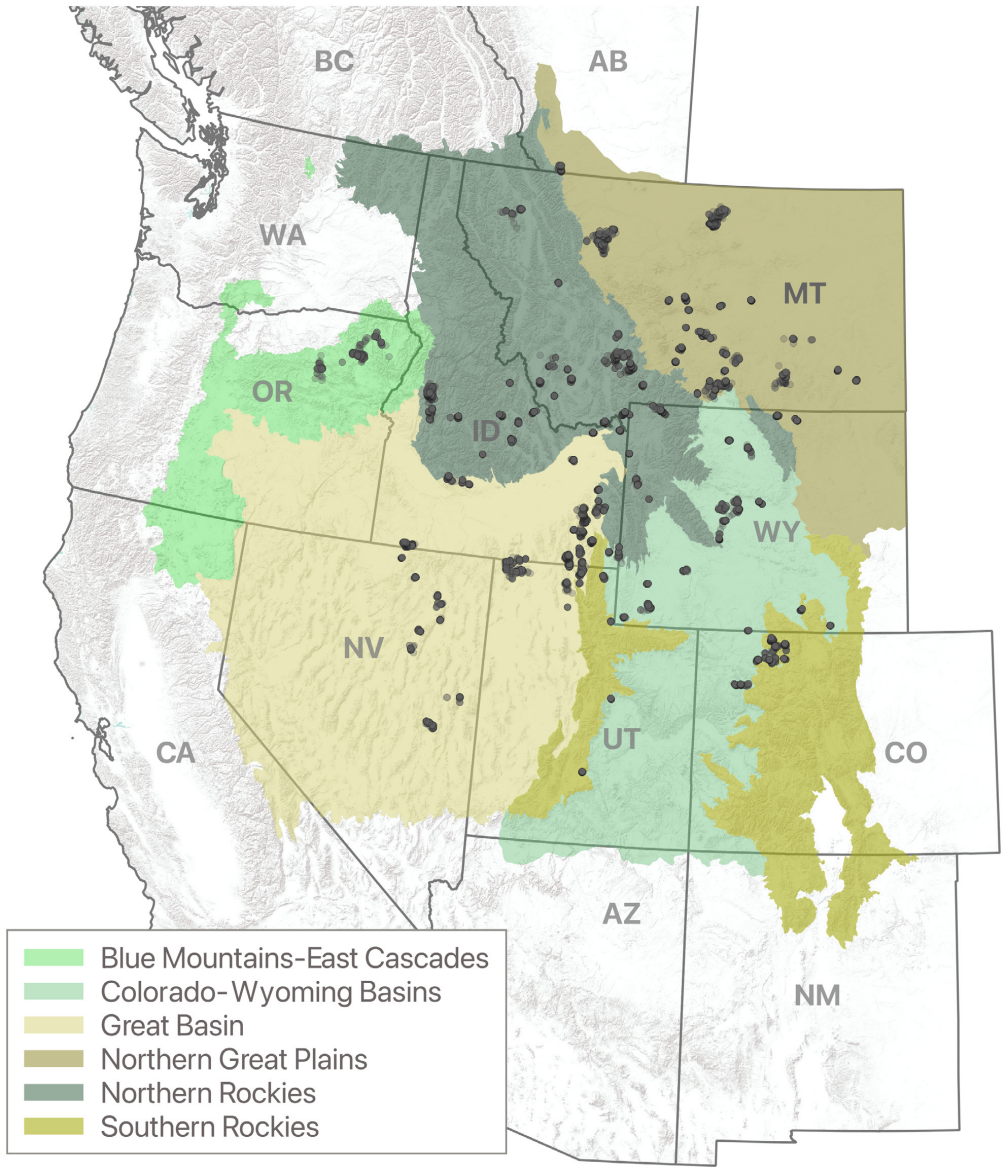
Study area extent defined by modified Level III ecoregions—shown in color. Points identify GPS summering sandhill crane locations collected 2014–2022.

While regional studies have provided insight into sandhill crane ecology (Littlefield et al., 1994; McWethy & Austin, 2009), high dispersal rates and relatively low densities during summering periods have limited our knowledge of landscape drivers structuring bird distributions. To address these information gaps, we marked 120 sandhill cranes with GPS tags and tracked their movements across eight U.S. states and Canadian provinces (Figure 1). A suite of remotely sensed ecological variables representing climate, human disturbance, land cover, and wetlands were then combined with bird locations in a cloud computing platform to model summer‐range sandhill crane distribution probabilities. Wetland variables identified as important predictors of bird distributions were evaluated in a post hoc analysis using satellite imagery to measure the long‐term (1984–2022) effects of climate‐driven surface water drying (Donnelly & King, 2020). Agricultural irrigation practices and land ownership were summarized within core sandhill crane summering areas as a measure of land‐use dependence. Study outcomes provide novel insight into the significance of wetland scarcity and flood‐irrigated agriculture in structuring sandhill crane summering distributions.

## 2 MATERIALS AND METHODS

### 2.1 Study area

The study area encompassed portions of the Intermountain West and Northern Great Plains, as defined by North American level three ecoregions (Wiken et al., 2011), including areas overlapping thirteen provinces and states in Canada (Alberta, British Columbia) and the United States (Arizona, California, Colorado, Idaho, Nevada, New Mexico, Montana, Oregon, Utah, Washington, and Wyoming). Ecoregional boundaries included known and suspected sandhill crane summering distributions based on published breeding surveys and GPS location data collected for this study (Conring, 2016; Drewien & Bizeau, 1974; Littlefield et al., 1994; Thorpe et al., 2022). Individual ecoregions were used to normalize summary results as a means to interpret patterns of land‐use and wetland change within the birds' range. Original ecoregional boundaries were largely unmodified but in some instances, were merged or clipped to align with sandhill crane distributions. Ecoregions within the Intermountain West included: (1) Blue Mountains‐East Cascades, (2) Colorado‐Wyoming Basins, (3) Great Basin, (4) Northern Rockies, and (5) Southern Rockies. The Northern Great Plains encompassed a single ecoregion (Figure 1).

Most of the study area is considered arid to semi‐arid, restricting wetlands to 1%–3% of the landscape footprint (Tiner, 2003). Water and wetlands are concentrated in valley bottoms along riparian floodplains. In the Northern Great Plains, glaciated pothole wetlands and small (<0.4 ha) human‐constructed ponds for livestock watering are common and can occur in high densities regionally. Riparian wetlands in the Intermountain West are heavily supported by flood‐irrigated grass‐hay production used for livestock forage production Donnelly et al. (2023). The practice diverts and spreads surface water from adjacent streams to irrigate perennial grasses that are seldom tilled and require relatively low chemical inputs (e.g., soil amendments, pesticides, and herbicides) compared to other more industrialized cropping practices. Publicly owned wetlands include lands administered by the U.S. Fish and Wildlife Service (USFWS), Environment Canada, and state wildlife agencies (hereafter ‘wildlife refuges’) managed to support sandhill cranes and other migratory waterbird populations. Other public wetland owners in the United States included the Bureau of Reclamation, the Bureau of Land Management, and the U.S. Forest Service.

The climate is characterized by cold winters and hot/warm summers. Wetland hydrology (i.e., flooding) is induced by local and high‐elevation snowmelt and runoff. In the Northern Great Plains, wetland hydrology can also be influenced by spring and summer precipitation. Most wetlands are inundated seasonally from early spring through mid‐summer, after which evaporative drying reduces surface water availability.

### 2.2 Crane captures and GPS deployment

Sandhill crane locations used to derive summering distributions were acquired from 120 individual birds captured and fitted with GPS tags. Tag deployments were partitioned among summering (*n* = 35) and wintering (*n* = 85) areas (See Appendix S1, Figure S1). Breeding status and sex of tagged adult sandhill cranes (*n* = 112) were unknown but assumed to have minimal influence on our broad‐scale assessment of summering distributions. Because sandhill cranes form lifelong pair bonds and maintain close contact with family groups throughout summering, migration, and wintering periods, space‐use was considered similar among sexes. Additionally, birds marked as juveniles (*n* = 8) and identified as non‐breeders exhibited movement and space‐use patterns similar to other marked birds in the study. Location acquisition rates of individual GPS tags varied from four to 45 points per day. Approximately 685,000 summering bird locations were collected from 2014 to 2022 (Figure 1), with over 85% of days containing seven or more acquisitions per 24 h. Detailed capture and GPS deployment procedures are provided by Collins and Grisham (2016) and Boggie and Collins (2018).

### 2.3 Defining summering locations

First passage time analysis (FPT) was used as a metric to differentiate sandhill crane summering and migration behaviors (Johnson & Wiens, 1992) by identifying spatiotemporal scales that birds interacted with landscapes (Fauchald & Tveraa, 2003). Because FPT is scale‐dependent, we calculated sandhill crane movement variance at radii from 1–100 km to distinguish slow localized summering movements from rapid long‐distance migration (sensu Le Corre et al., 2014). We segmented FPT results temporally using Behavioral Change Point Analysis (BCPA; Lavielle, 1999; Lavielle & Teyssière, 2006). This method optimized segmentation of seasonal space‐use by minimizing a contrast function (i.e., a function measuring the discrepancy between rapid long‐distance migration and an underlying model characterized by slow localized movements). We applied BCPA using a mean contrast function and minimum location use parameter of 10. Differences in GPS acquisition rates among birds did not influence BCPA segmentation due to large‐scale migration versus localized movements that were distinguished. Both FPT and BCPA were implemented with R‐package adehabitatLT (Calenge, 2011).

Movement segments were classified for individual birds using a rule‐based approach linked to seasonal timing and duration of unique space‐use patterns. Summering segments were made of bird locations associated with prolonged localized movements beginning in early spring and ending in late summer. Migration segments were classified as rapid long‐distance movements occurring on either end of summering periods. All classified BCPA results were exported to a GIS for visual inspection and editing to ensure classifications aligned with observed bird movements. Summering locations were then combined as a representative sample of all individual birds sampled.

### 2.4 Landscape variables

We used a suite of 15 continuous spatially explicit landscape variables to model summering sandhill crane distributions (Table 1). To fill data gaps in available wetland data, we developed novel surface water models to depict the timing and duration of wetland flooding (hereafter “wetland hydroperiod”), a key delimiter of vegetative structure and foraging resources associated with waterbird use (Foti & Del Jesus, 2012). Following methods outlined by Donnelly and King (2021), we mapped monthly patterns of wetland inundation for all wetland and riparian systems within the study area. Measurements were derived from surface reflectance Landsat 8 and 9 Operational Land Imager satellite imagery using a 30 × 30 m pixel grid to capture hydroperiod diversity within individual wetlands. Heavily forested wetlands were omitted from our analysis due to ocular masking from tree canopy, making it difficult to measure surface water conditions. Hydroperiods were summarized by totaling the number of months individual pixels were flooded from January to December. Wetlands were then classified as ‘temporary’ (flooded <2 months), ‘seasonal’ (flooded >2 and <9 months), or ‘semi‐permanent’ (flooded >8 months) using standards similar to Cowardin et al. (1979). This process was replicated annually from 2014–2022 to coincide with sandhill crane GPS data collection periods. Large deep water bodies (e.g., reservoirs and large rivers) were omitted from our analysis to remove bias from wetland features that do not directly support sandhill crane summering habitat. A detailed description of methods used to generate wetland hydroperiods is provided in Appendix S1.

**TABLE 1:** List of variables used to predict sandhill crane summering distributions.

Variable	Source
Wetlands	
All wetland density^a^	Satellite‐derived, see supplemental material
Temporary wetland density^a^	Satellite‐derived, see supplemental material
Seasonal wetland density^a^	Satellite‐derived, see supplemental material
Semi‐permanent wetland density^a^	Satellite‐derived, see supplemental material
Wetland diversity index^a^	Satellite‐derived, see supplemental material
Landcover	
Annual grass and forb cover^a^	Allred and Bestelmeyer (2021)
Bare ground cover^a^	Allred and Bestelmeyer (2021)
Perennial grass and forb cover^a^	Allred and Bestelmeyer (2021)
Shrub cover^a^	Allred and Bestelmeyer (2021)
Tree cover^a^	Allred and Bestelmeyer (2021)
Late summer productivity^a^	Satellite‐derived, see supplemental material
Human disturbance	
Global human modification layer (2016)	Kennedy and Oakleaf (2019)
Road density	https://www.census.gov/
Climate	
Frost‐free days^a^	Thornton et al. (2022)
Landform	
Slope	https://www.usgs.gov/

^a^ Mean 2014–2022.

We applied a mean focal filter to wetland distributions to estimate densities within landscapes as a means to capture the gradient of space‐use sandhill cranes exhibited around these features. The kernel diameter used for focal calculations (~3 km) was equivalent to the eightieth percentile home range estimates for summering sandhill cranes to ensure outputs encompassed bird movements. The percentile break was identified by reviewing ranked order estimates of sandhill crane home range size. While this approach was somewhat subjective, it made it possible to estimate the upper extent of bird movements while removing outliers. Home range estimates (*n* = 120) were derived for individual birds from 2014 to 2022 using GPS locations and minimum convex hull calculations. Wetland density measures were replicated for all wetland hydroperiods combined and individually for each hydroperiod class. We accounted for wetland diversity as a factor influencing sandhill crane distributions by applying Simpon's index of diversity (Simpson, 1949):

$$D=1-\frac{\sum n\left(n-1\right)}{N\left(N-1\right)}$$

where *n* = the area of each wetland hydroperiod class (temporary, seasonal, or semi‐permanent) and *N* = the total number of wetland hydroperiod classes (temporary, seasonal, semi‐permanent). A mean focal filter approach previously described was used to estimate wetland diversity within the median home range of summering sandhill cranes.

All wetland hydroperiod and wetland diversity measures presented were calculated as an average during nesting and early colt‐rearing periods (April 1 to June 30) for each year GPS locations were acquired (2014–2022). Results were linked with annual bird locations to align resource conditions with space‐use over time.

### 2.5 Crane distribution modeling

We modeled the relationship between sandhill crane GPS locations and environmental factors to predict summering distributions using a Random Forest regression algorithm (Breiman, 2001). Random Forest uses machine learning to produce predictive models that account for nonparametric and highly complex ecological interactions (Mi & Huettmann, 2017). This modeling approach is less sensitive to collinearity issues among predictors while remaining robust to overfitting Culter et al. (2007). Hyperparameter tuning was used to identify the optimal number of trees needed (*n* ~ 4000) to maximize model accuracy (Oshiro et al., 2012). Because sandhill cranes exhibited constrained movement patterns with clustered space‐use in small areas due to nesting and early colt‐rearing behaviors, locations were sampled to remove bias from spatial autocorrelation when formatting Random Forest training data. A total of 20,000 locations from 685,000 GPS points collected were selected randomly using a rule‐based process that restricted the selection of locations within a distance of 400 m from other selected points. Pseudo‐absence locations needed for model training were generated within the study area and combined with sampled sandhill crane GPS locations (*n* = 20,000). We optimized Random Forest performance that can be sensitive to uneven sampling among classes by maintaining a balanced ratio of presence‐absence locations (Liu et al., 2019).

To reduce the potential of false absences, GPS sandhill crane locations were buffered by 1.5 km to exclude pseudo‐absence points from sandhill crane use areas (VanDerWal & Shoo, 2009). Buffered distance approximated the size of the median sandhill crane summer home range area, as described previously. The rfUtilites package in R (Evans & Murphy, 2015) was used to test and remove multicollinear variables. All variables were retained. To assess variable importance in Random Forest, we used the sum of squared errors measured as the total increase in node purity resulting from variable splitting averaged over all trees in the model. Partial dependence plots for variables were developed to depict the marginal effects influencing the propensity of sandhill crane resource selection.

A holdout ratio of 20% (*n* ~ 8000) was randomly selected to evaluate model performance using *k*‐fold (*k* = 5) cross‐validation and a relative classification probability threshold of ≥0.65. Classification probability thresholds and dispersal constraints were implemented through expert opinion conducted by regional state and provincial wildlife biologists (*n* > 25) familiar with local sandhill crane distributions (Hengeveld & Haeck, 1982). Thresholding aimed to identify “core” sandhill crane summer range representative of bird use patterns clustered around scarce wetlands and riparian resources. While partly subjective, this approach made it possible to incorporate regional context into model outcomes, potentially increasing relevance to science end users (Sofaer & Jarnevich, 2019) and creating a spatial framework for prioritizing conservation in high‐use potential (hereafter “core”) summering areas. In addition, feedback from this process was used to inform model parameters to remove areas of unoccupied habitat on the fringe of the bird's predicted summer range. All model results were interpreted using a core area approach that summarized bird use and landscape variables within core (i.e., classification relative probability thresholds ≥0.65) summer use areas.

### 2.6 Wetland trends

High‐ranking wetland variables identified by the Random Forest model were applied to a post hoc analysis using Landsat satellite imagery to measure long‐term (1984–2022) wetland hydrology in sandhill crane core summering areas. Following methods outlined by Donnelly and King (2020), surface water patterns were monitored annually as three‐month means that coincide with sandhill crane nesting and early colt‐rearing (Apr–Jun). Increased surface water area during this time correlates positively to sandhill crane nest success (Austin et al., 2007). Analyses were binned by hydroperiod (i.e., temporary, seasonal, and semi‐permanent) to examine change within wetland factors used to predict sandhill crane distributions. Wetland hydroperiod classes were modeled using mean conditions during the sandhill crane GPS data collection period (2014–2022) and were held constant over time. Trends were measured by fitting Kendall's Tau‐b rank correlation to pixel surface water change through time. Trend measurements were partitioned eco‐regionally to isolate spatial differences in hydrologic function (Lauenroth et al., 2014).

### 2.7 Land‐use

Core areas were summarized by private, public, and tribal land management designations to measure crane land‐use reliance. Private land‐use was categorized as (1) lands protected under conservation easement, (2) flood‐irrigated grass‐hay cultivation (hereafter “grass‐hay”), or (3) other private lands. Public lands were partitioned by agencies (e.g., Bureau of Land Management, Forest Service). Tribal lands were denoted as (1) grass‐hay or (2) other tribal lands. Public and protected lands were defined using the Protected Area Database for the United States and the Canada Lands Digital Cadastral Dataset. Grass‐hay was defined using Donnelly et al.'s (2023) grass‐hay production layer. This dataset was chosen because of the strong spatial correlation between grass‐hay delineations and riparian floodplains—known to support flood‐irrigated wetlands (sensu Gordon & Paige, 2020) and summering bird distributions in portions of their range (McWethy & Austin, 2009). Because wetland hydrology was identified as a key component in structuring sandhill crane summering distributions, wetland hydroperiods were also summarized by private, public, and tribal land management designations.

### 2.8 Data processing

All image processing and raster‐based analyses were conducted using Google Earth Engine, a cloud‐based geospatial processing platform (Gorelick & Hancher, 2017). All GIS analyses were performed using QGIS (QGIS Development Team, 2020). Plotting and statistical analyses were generated using the R environment (R Core Team, 2019; RStudio Team, 2019), including R‐packages not previously mentioned; randomForest (Liaw & Wiener, 2002), and Tidyverse (Wickham & Averick, 2019).

## 3 RESULTS

### 3.1 Summering distributions

**FIGURE 2:**
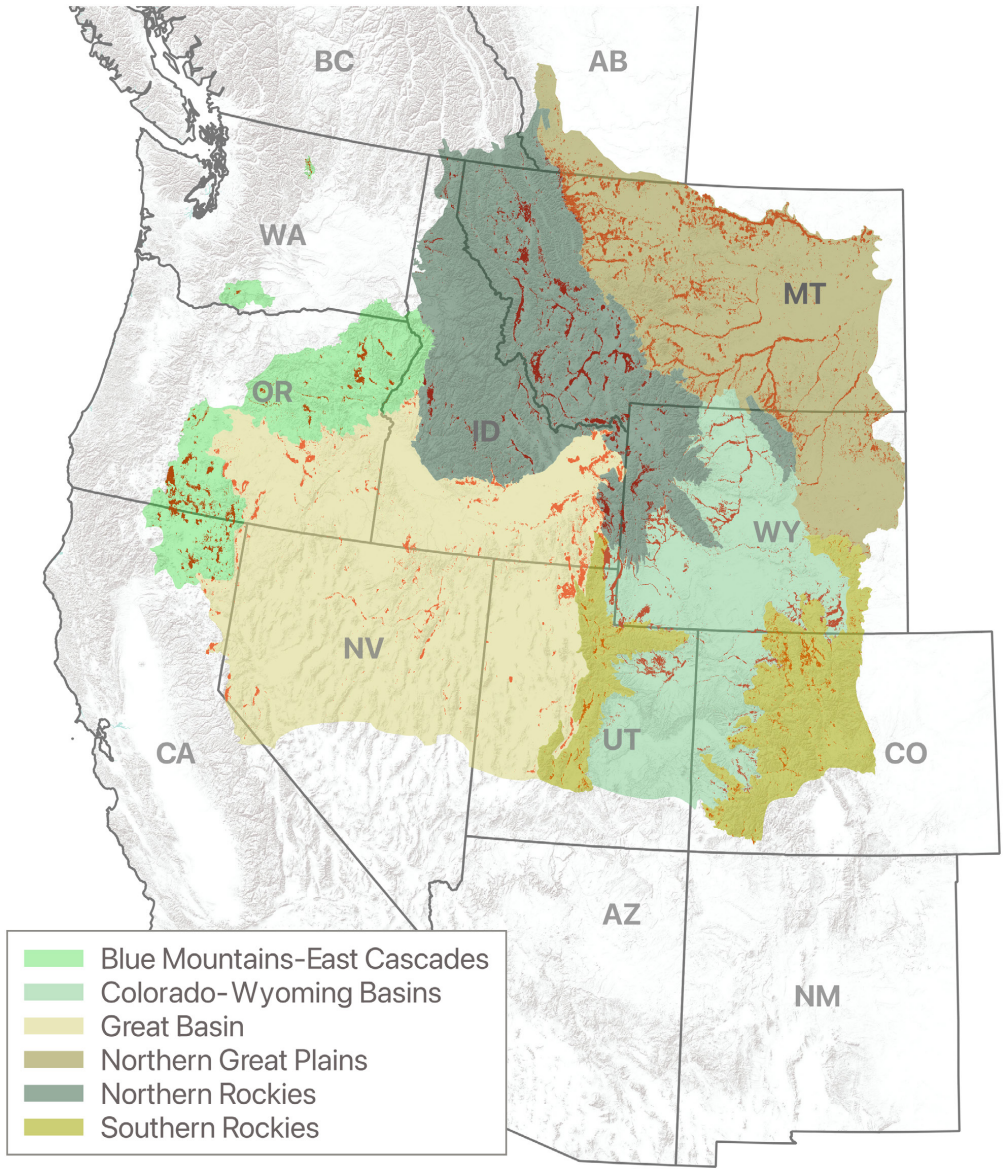
Sandhill crane core summering distributions shown in red as occurrence probabilities ≥65%. Modified ecoregional polygons encompass estimated summer range extent.

Core sandhill crane summering areas accounted for ~5% (7.6 million ha) of the total estimated range (~145 million hectares) in western North America, encompassing eleven states and provinces in the U.S. and Canada (Figure 2). Eastern portions of the Great Plains ecoregion and southern portions of the Colorado‐Wyoming Basins, Great Basin, and Southern Rockies ecoregions were modified to represent occupied sandhill crane range. Northern areas of the Northern Rockies ecoregion were also modified (Figure 2). The percentage of core area was greatest in the Northern Great Plains, containing 34.5% of the total core area, followed by the Northern Rockies (19.1%), Great Basin (15.7%), Colorado‐Wyoming Basins (14.8%), Southern Rockies (8.0%), and Blue Mountain‐East Cascades (7.8%) ecoregions. By state, Montana accounted for 41.6% of core area, followed by Wyoming (16.3%), Idaho (12.8%), Oregon (7.9%), Utah (7.4%), Colorado (7.0%), Nevada (2.9%), California (2.6%), and Washington (0.2%). Canada comprised less than 2.0% of the core area distribution located in southwest Alberta and southeast British Columbia. The model mean square error for *k*‐fold cross‐validation (*k* = 5) was 0.29, suggesting that the model reasonably estimated sandhill crane potential use probabilities from a statistical perspective.

### 3.2 Landscape variables

**FIGURE 3:**
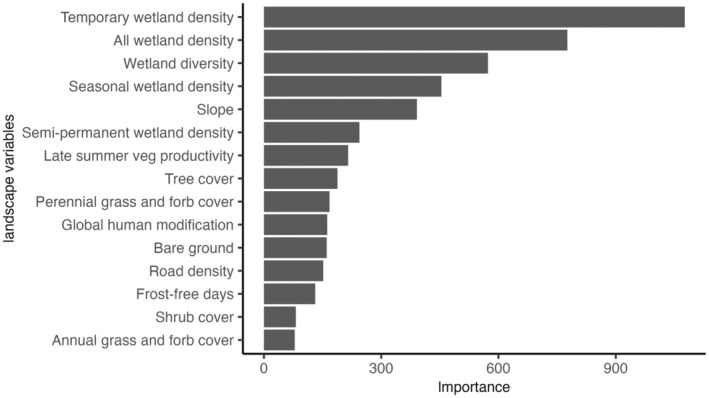
Random Forest variable importance for sandhill crane distribution predictions measured as the total increase in node purity resulting from variable splitting, averaged over all trees.

**FIGURE 4:**
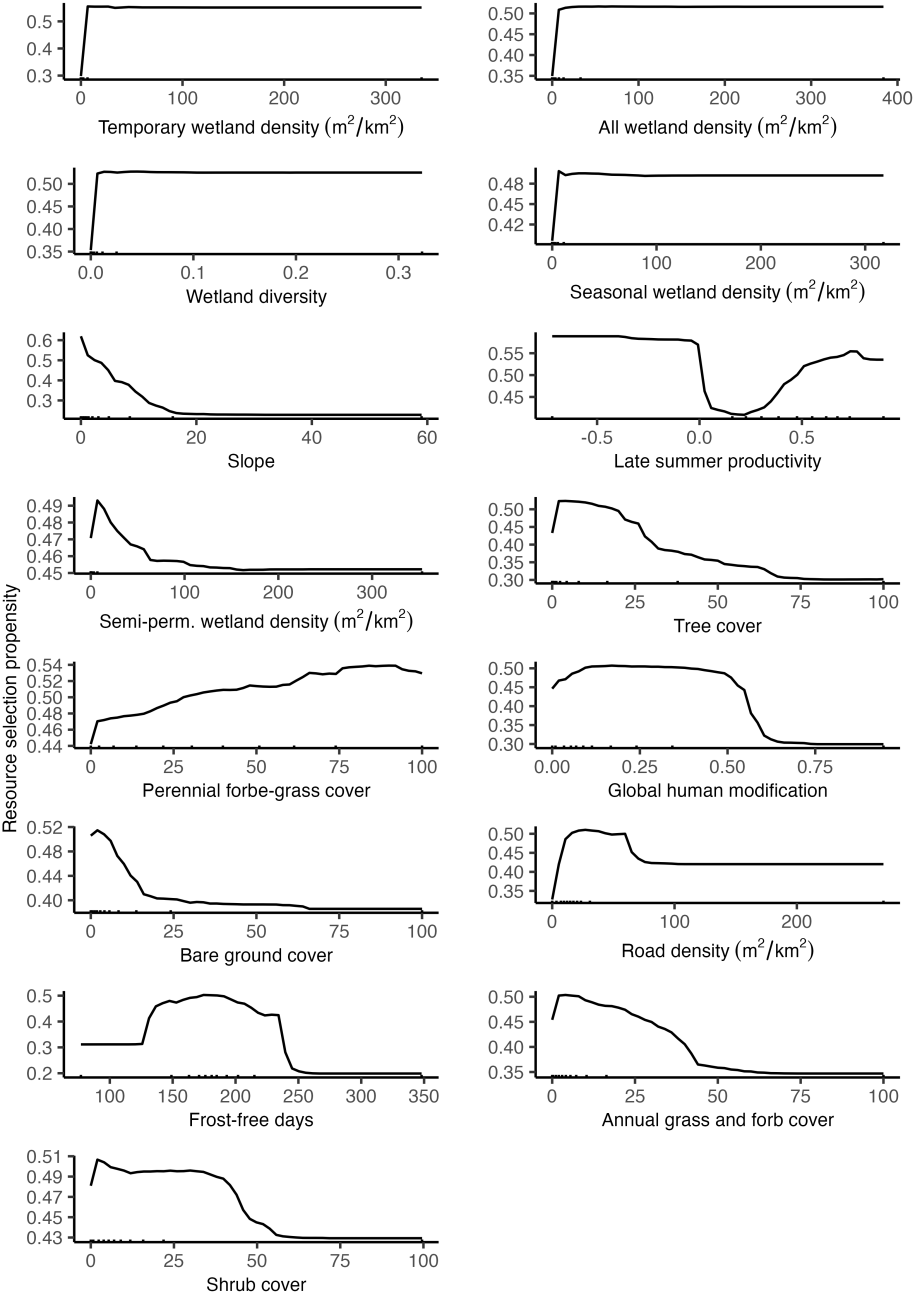
Partial dependence plots depicting marginal effects as a measure of resource selection propensity for each landscape variable used in Random Forest model predictions of sandhill crane summering distributions.

Sandhill crane summering distributions were structured primarily by wetland density, wetland diversity, and slope (Figure 3). Resource selection propensity for temporary wetland density, overall wetland density, wetland diversity, and seasonal wetland density increased sharply at measures above zero and was maintained as measures increased (Figure 4). Sandhill crane occurrence probabilities were negatively correlated to slope and declined to near zero in areas greater than 20%. Other high‐ranking variables included semi‐permanent wetland density and late summer vegetative productivity. Bird probabilities were positively correlated to semi‐permanent wetlands at low densities and negatively correlated to those >30 m^2^/km^2^. Sandhill crane relationships to late summer vegetative productivity were bimodal, showing high correlations to NDVI values <0 and >0.2. This pattern was likely caused by bird use of shallow temporary and seasonal wetlands that typically have negative NDVI values due to the absorption of near‐infrared wavelengths by water. Human disturbance measures (i.e., road density and global human footprint) were negatively correlated to sandhill crane occurrence probabilities but were not as important to shaping bird distributions when compared to other variables. Sandhill crane summering areas were generally associated with areas between 125 and 250 frost‐free days. Tree cover and bare ground were negatively correlated with occurrence. Shrub, annual grass, and forb cover were least important to structuring sandhill crane summering distributions.

### 3.3 Land ownership

**FIGURE 5:**
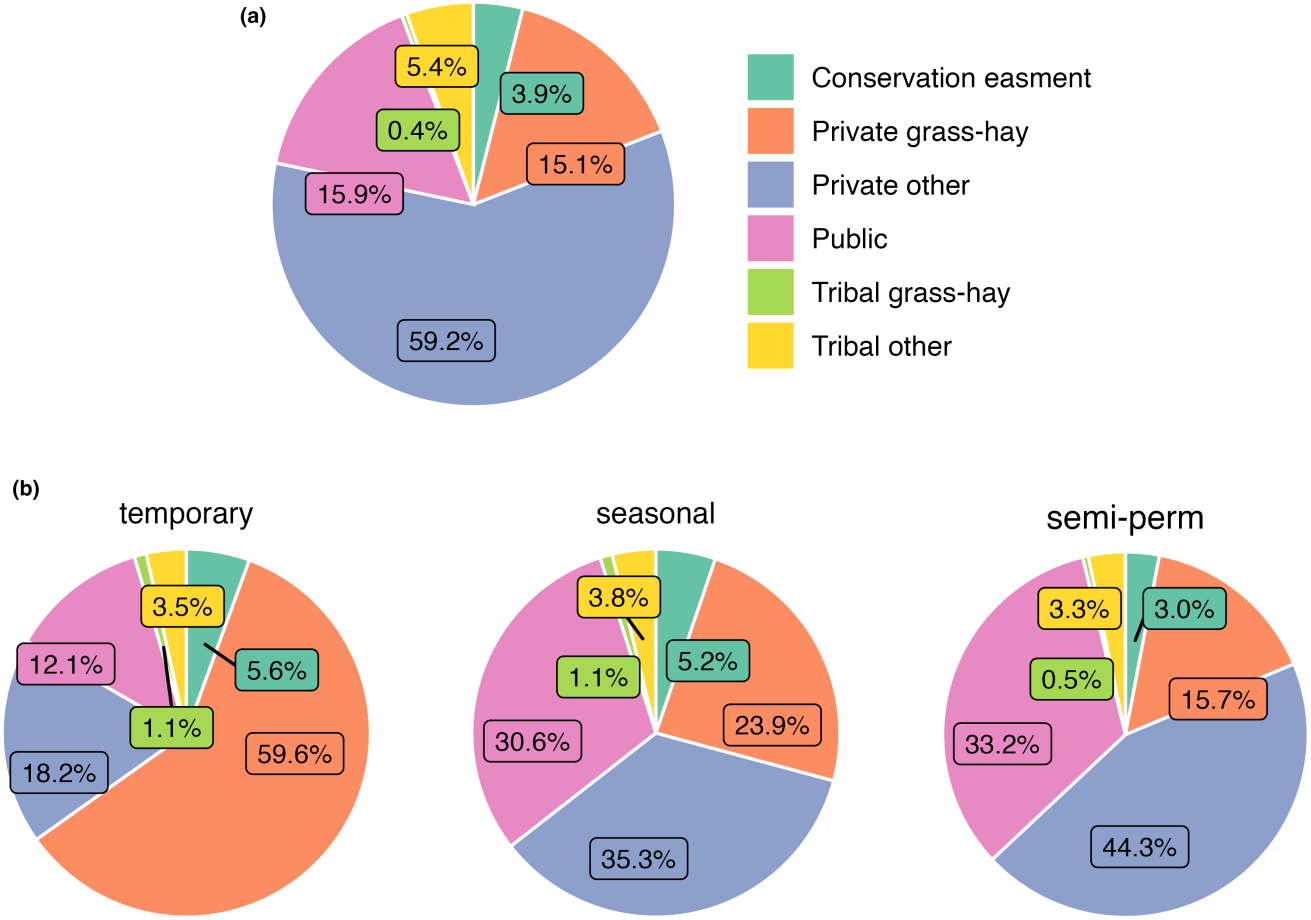
Proportional land ownership, land‐use (a), and wetland distributions (b) in sandhill crane core summering areas. Wetlands are summarized by hydroperiod classes (temporary, seasonal, and semi‐permanent).

Only 40.3% of sandhill crane summer range was privately owned but it accounted for approximately 78% of the range in core summering areas (Figure 5). In the Intermountain West, core summering areas overlapped 93% of grass‐hay agricultural practices. Public lands accounted for approximately 16% of core summering areas, most of which were administered by the U.S. Forest Service, the Bureau of Land Management, and the U.S. Fish and Wildlife Service (see Appendix S2, Table S1). Tribal nations, including areas of Tribal grass‐hay cultivation, accounted for approximately 6% of core summering areas, primarily on the lands of Blackfeet, Shoshone‐Paiute, Eastern Shoshone, and Northern Arapaho peoples (Figure 5).

Ownership and land‐use practices associated with core area wetland features differed from overall ownership patterns (Figure 5). Core areas encompassed 733,123, 574,654, and 330,512 ha of flooded temporary, seasonal, and semi‐permanent wetlands. Privately owned grass‐hay production supported 59.6% of temporary wetlands, while an additional 18.2% and 12.1% were attributed to other private and public lands. Seasonal wetlands were distributed relatively evenly between public, private, and grass‐hay lands, accounting for 30.6%, 35.3%, and 23.9% of abundance. Private and public lands accounted for over three‐quarters of semi‐permanent wetlands, representing 44.3% and 33.2% of abundance while grass‐hay production on private lands accounted for 15.7% of the total. The U.S. Fish and Wildlife Service, U.S. Forest Service, State Wildlife Agencies, and the Bureau of Land Management accounted for most publicly owned temporary, seasonal, and semi‐permanent wetlands (see Appendix S2, Tables S2–S4). Combined Tribal lands (including associated grass‐hay) and conservation easements accounted for approximately 1%–5% of temporary, seasonal, and semi‐permanent wetlands within core areas.

### 3.4 Wetland trend

**FIGURE 6:**
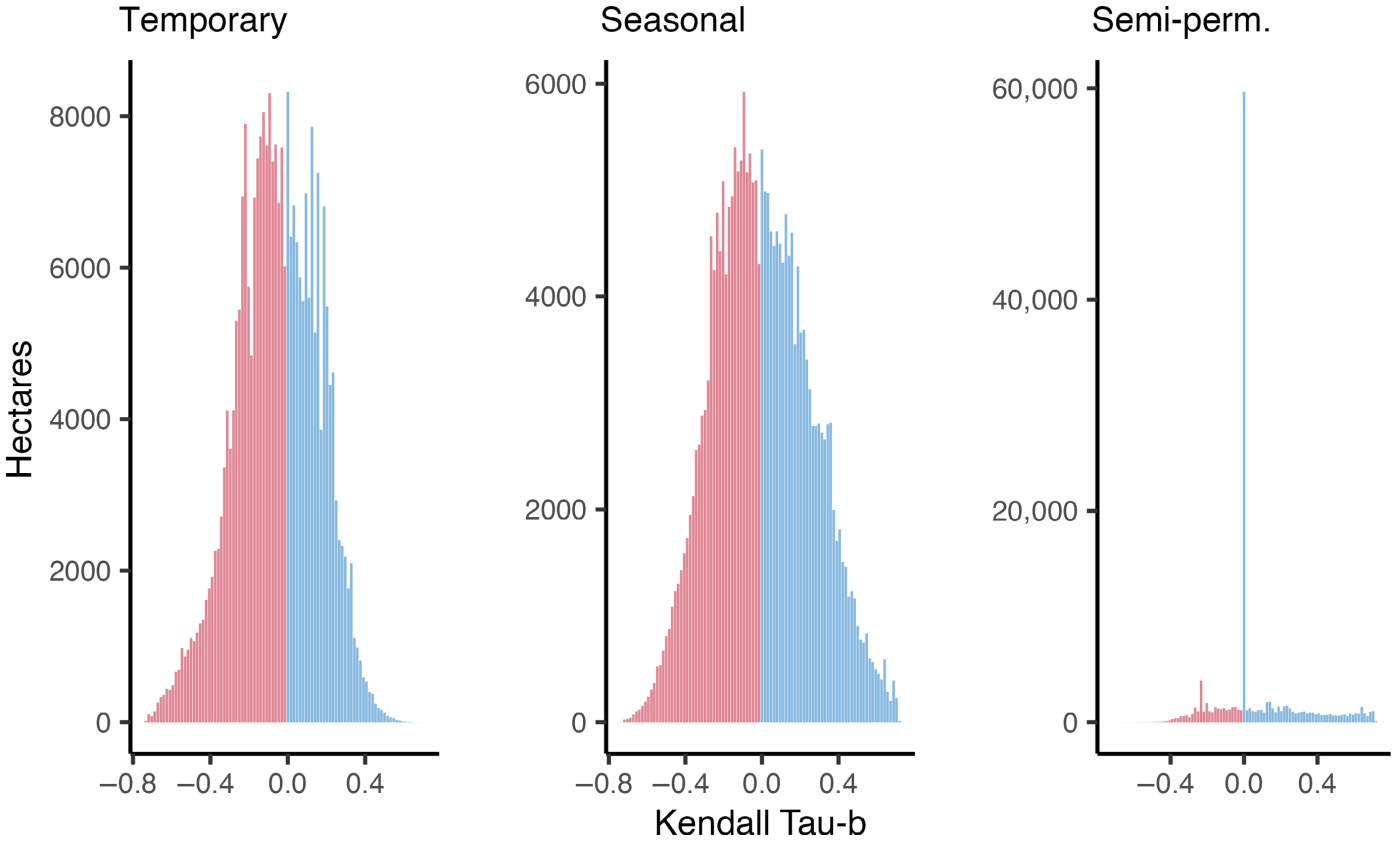
Kendall's Tau‐b rank correlation distributions by wetland hydroperiod class. Summaries represent all wetlands within sandhill crane core areas. Negative values indicate wetland drying associated with reduced duration or frequency of flooding since 1984.

Wetlands accounted for only for 1.2% of sandhill crane summer range. Wetland surface water monitoring in core areas during sandhill nesting and early colt‐rearing periods (April 1 to June 30) showed drying trends correlated to hydroperiod ephemerality. Areas of negative Kendall's Tau‐b rank correlation (i.e., reduced duration or frequency of flooding) were greatest in temporary wetlands and increasingly less pronounced in wetlands characterized by seasonal and semi‐permanent hydrologies (Figure 6). Overall Kendall's Tau‐b summaries showed a majority of temporary and seasonal wetlands trending toward dryer states. Rates of change within ecoregions were variable with drying most pronounced in the Great Basin, which showed 71.3% and 65.5% of temporary and seasonal wetlands trending toward dryer states since 1984 (Table 2). The Northern Rockies, Southern Rockies, and Blue Mountain‐East Cascades ecoregions also experienced drying in over 50% of temporary wetland areas, indicating long‐term declines in hydrologic function. The Colorado‐Wyoming Basins and the Northern Great Plains showed stable to wetter trends across all wetland hydrologies. Semi‐permanent wetlands within core areas remained relatively stable across all ecoregions, with most wetlands exhibiting Kendall's Tau‐b measures ≥0. See Appendix S2, Figures S2–S7 for Kendall's Tau‐b rank correlation distributions by ecoregion.

**TABLE 2:** Proportion of wetland area drying within sandhill crane core areas as determined by negative Kendall's Tau‐b rank correlation values.

	Temporary	Drying	%drying	Seasonal	Drying	%Dryng	Semi‐perm	Drying	%Drying
Blue Mountains–East Cascades	26,966	16,004	59.3%	49,332	24,963	50.6%	22,708	7642	33.7%
Colorado–Wyoming Basins	86,003	38,883	45.2%	38,662	13,380	34.6%	36,717	2653	7.2%
Great Basin	68,296	48,678	71.3%	57,601	37,729	65.5%	39,087	9545	24.4%
Northern Great Plains	32,423	15,901	49.0%	40,239	13,464	33.5%	55,136	6451	11.7%
Northern Rockies	50,978	34,808	68.3%	52,951	21,520	40.6%	30,510	2021	6.6%
Southern Rockies	21,537	12,921	60.0%	11,183	4334	38.8%	6697	405	6.0%

*Note*: Summaries are partitioned by ecoregion and wetland hydroperiod. Wetland conditions were measured for each year from 1984 to 2022 during sandhill crane sandhill nesting and early colt‐rearing periods (April 1 to June 30).

## 4 DISCUSSION

Our analysis provides a comprehensive and updated estimate of sandhill crane summer range in the American West. The results of this study fill an essential gap in our understanding of bird distributions and the role wetland scarcity and agriculture play in structuring sandhill crane occurrence. Although wetlands accounted for 1.2% of their summer range, they were key predictors of occurrence probabilities. Core areas were structured primarily by temporary wetlands supported by grass‐hay agriculture concentrated in riparian floodplains Donnelly et al., 2023.Wetland drying observed in core areas from 1984 to 2022 was consistent with climate‐induced trends observed in regional studies (Donnelly et al., 2022; Donnelly & King, 2020), representing an emerging ecological bottleneck that could limit sandhill crane summering range in the future.

Our work indicates a significant sandhill crane range expansion into the Northern Great Plains. Drewien and Bizeau's (1974) original range estimations for the Rocky Mountain population segment documented historical eastward movement in 1972 with the furthest east‐known breeding pair in west‐central Wyoming near Ocean Lake. Over the past 50 years, we show core summering area expansion of 350 km east and 650 km north, encompassing nearly the entire rangeland ecosystem east of the Rocky Mountains in Montana and Wyoming, including portions of Alberta, Canada. Annual surveys conducted from 1996 to present identify moderate long‐term population growth that is attributed primarily to a 240% and 210% increase in sandhill crane abundance in Montana and Wyoming (Thorpe et al., 2022), affirming Northern Great Plains range expansion identified by our analysis. Hunting pressure targeting lesser sandhill cranes (*Antigone canadensis canadensis*) in the mid‐continent population may act as a potential barrier to further eastward expansion due to incidental harvest, preventing the establishment of breeding pairs (Krapu & Brandt, 2010). Despite a gap in sandhill crane location data in some western portions of the summering range (i.e., California, Nevada, Oregon, and Washington), our core area model aligned well with regional surveys conducted in the mid‐1990s by Littlefield et al. (1994) and Rawling (1992), as confirmed by our independent review from regional wildlife managers. Additionally, our results suggest that outside the Great Plains, bird distributions have remained relatively stable within their previously known summer range (Drewien & Bizeau, 1974; Littlefield et al., 1994; Rawling, 1992).

At broad scales, wetland density and diversity were the primary factors underpinning core area distributions, partly dismissing the notion that human disturbance acts as a mechanism to limit occurrence during the breeding period (Armbruster, 1987; Drewien & Bizeau, 1974). While variable importance scores for ‘road density’ and ‘global human disturbance’ were low, resource selection propensity by sandhill cranes suggests a relatively high tolerance for these conditions. Early observations describing sandhill crane habitat suitability were confined mainly to National Wildlife Refuges that provided birds with isolated nesting opportunities and may have biased interpretations of their need for seclusion during breeding (Armbruster, 1987; Drewien & Bizeau, 1974). Sandhill crane range expansion identified here may be an indicator of changing density dependence in core summering areas that has reduced the availability of isolated breeding locations, forcing birds to rely on alternative resources. Because this study did not examine sandhill crane vital rates, it was unclear if bird tolerance for human disturbance functions as a biological sink, where adaptive plasticity for marginal environmental conditions results in reduced nest success and colt survival. Slow life history traits relative to other waterbirds (i.e. low annual fecundity and high adult survival) in sandhill cranes may also mask detectability of these behavioral and habitat interactions at the population level (Drewien et al., 1995).

Our model depictions of declining surface water likely reflect a new normal in wetland conditions that could impact resource availability in core sandhill crane summer range, particularly in the Intermountain West's Great Basin and Northern Rockies ecoregions where over two‐thirds of temporary wetlands experienced long‐term drying. Wetlands play a crucial role in sandhill crane nesting ecology as evidenced by their attraction to water and its positive role in nest survival (McWethy & Austin, 2009). Corroborating our evidence of wetland scarcity for future nesting populations are climate projections showing a 1–3°C increase in Great Basin summer temperatures by 2020–2050 (Snyder & Evers, 2019). Increasing temperatures across the Intermountain West are predicted to decrease snowpack runoff supporting wetland hydrology while simultaneously increasing agricultural water needs through elevated evaporative demand from crops (Elliott & Deryng, 2014; Mix et al., 2009).

Sandhill cranes' symbiotic relationship with private lands agriculture is foundational to structuring this species' summer distribution, as evidenced by core summering areas overlaying 93% of flood‐irrigated grass‐hay production in the Intermountain West. Our results suggest increasing water scarcity due to warming temperatures and prolonged drought events are the primary threat to maintaining flood‐irrigation practices supporting summering birds. Growing urban water demands may also compound projected climate shortfalls by negatively impacting irrigated agriculture in some core areas (Schaible & Aillery, 2017). Efforts in Colorado and Nevada, for example, have proposed agricultural fallowing through the purchase and repurposing of rural irrigation water for municipal use (Thorvaldson & Pritchett, 2006; Welsh & Endter‐Wada, 2017). Such scenarios frequently require out‐of‐basin water transfers, reducing local wetland availability supported through irrigation while eliminating local ecosystem services benefiting climate resilience in riparian ecosystems (Blevins et al., 2016; Zhuang, 2016). Additionally, loss of irrigation can increase subdivision risk that removes wildlife‐compatible land‐use practices in rural landscapes as producers sell off land for development due to its reduced agricultural value (Dozier & Arabi, 2017).

**FIGURE 7:**
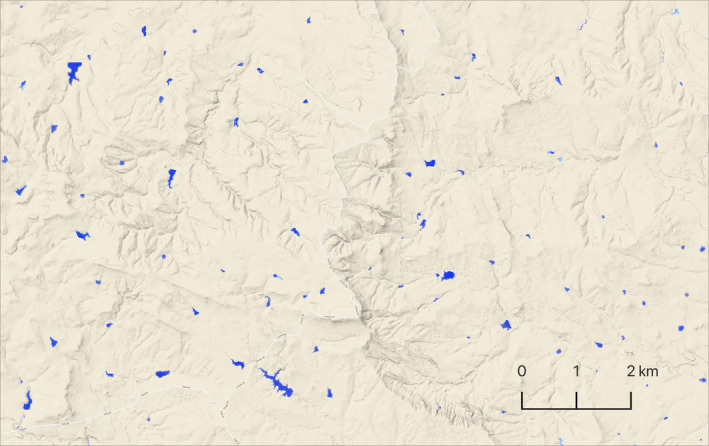
Stock pond density example from Northern Great Plains, Southeast Montana. Surface water is shown in blue.

In the Northern Great Plains, wetland resilience and sandhill crane range expansion may in part be an ecosystem service provided by agriculture. Ketchum et al. (2023) found irrigated agriculture in the region is primarily confined to river floodplains, promoting groundwater recharge by growing crops over highly permeable soils, allowing the return of unconsumed water to the system. We highlight similar ecosystem services tied to riparian (flood‐irrigated) grass‐hay production in the Intermountain West (Gordon & Paige, 2020; Kendy & Bredehoeft, 2006); however, this practice represents less than 3% of irrigated lands regionally Donnelly et al., 2023, the remainder of which is supported by out‐of‐floodplain surface water diversions or groundwater pumping that may have limited groundwater recharge or return flow benefits. Additionally, high densities of small (<0.4 ha) ponds constructed for livestock watering, evident in our wetland mapping data (Figure 7), may have offset patterns of landscape drying and increased surface water availability in rangeland ecosystems that previously lacked natural wetlands to sustain summering crane populations. While our analysis does not directly quantify stock pond abundance, their documented proliferation in the Great Plains region has been identified as a driver of increased biodiversity in agricultural landscapes (Swartz & Miller, 2021).

Tribal lands accounted for a relatively small proportion, approximately 6%, of sandhill crane core area but included sites of relatively high ecological value for migratory waterbirds. Core area distribution on the Fort Hall Reservation lands of the Shoshone‐Bannock Tribes (Great Basin ecoregion), for example, is also recognized as a regionally significant shorebird migration site supporting 50,000–80,000 individuals and thirty species annually (Senner et al., 2016). Likewise, core area distribution on the Wind River Reservation lands of the Eastern Shoshone and the Northern Arapaho Tribes (Colorado‐Wyoming Basins ecoregion) also support important spring and fall migration staging locations for sandhill cranes summering across the eastern half of their range (Donnelly & King, 2021). Senior water rights often associated with tribal lands (Sanchez et al., 2023) may elevate their importance to sandhill cranes and other wetland‐dependent wildlife as projected climate scenarios indicate increasing water scarcity across most of the summering range. Increasing Tribal engagement can also provide opportunities to integrate traditional ecological knowledge into broader conservation strategies that may benefit from the perspective of indigenous stewardship (Leonetti, 2010).

From a broader ecosystem perspective, our findings suggest sandhill cranes function as a unique surrogate species for agroecology and climate change adaptation strategies considerate of wetland‐dependent wildlife and solutions for improved water security (sensu Caro & O'Doherty, 1999). Intrinsic linkages between wetlands and agricultural ecosystem services, represented by our bird distributions in the Intermountain West and Northern Great Plains, support a spatially explicit conservation framework capable of targeting maintenance of beneficial flood‐irrigation practices (i.e., grass‐hay) while implementing adaptive measures for out‐of‐floodplain cultivation that reduces overall water use through maximized efficiency. Moreover, sandhill cranes have a long heritage of cultural values in western North America, supporting regional festivals celebrating their seasonal arrivals that have become important drivers in rural economies—making these birds ideal ambassadors for sustainable wetland ecosystems in agricultural landscapes. The cultural significance of sandhill cranes extends to many Native American Tribes that have revered these birds for centuries (Johnsgard, 2017). To inform conservation design, we make our wetland and sandhill crane core summering data publicly available as interactive web‐based mapping tools. We encourage the use of our results to inform conservation solutions through collaborative and proactive decision‐making among local and regional stakeholders while considering the social, ecological, and economic factors of conservation and management actions at local scales.

## AUTHOR CONTRIBUTIONS

**J. Patrick Donnelly:** Conceptualization (lead); data curation (lead); formal analysis (lead); funding acquisition (lead); investigation (lead); methodology (lead); project administration (lead); resources (lead); software (lead); supervision (equal); validation (lead); visualization (lead); writing – original draft (lead); writing – review and editing (lead). **Daniel P. Collins:** Conceptualization (supporting); data curation (equal); formal analysis (supporting); investigation (supporting); methodology (supporting); writing – review and editing (equal). **Jeffrey M. Knetter:** Data curation (equal); funding acquisition (equal); project administration (supporting); resources (equal); writing – review and editing (equal). **James H. Gammonley:** Conceptualization (supporting); funding acquisition (equal); investigation (supporting); validation (equal); writing – review and editing (equal). **Matthew A. Boggie:** Data curation (equal); investigation (supporting); project administration (supporting); writing – review and editing (equal). **Blake A. Grisham:** Data curation (equal); project administration (supporting); writing – review and editing (equal). **M. Cathy Nowak:** Data curation (equal); project administration (supporting). **David E. Naugle:** Conceptualization (supporting); investigation (supporting); project administration (equal); writing – review and editing (equal).

## CONFLICT OF INTEREST STATEMENT

The authors declare no conflicts of interest.

## Supporting information


Appendix S1.

Appendix S2.

## Data Availability

Data visualization and interactive mapping tools are available here.

## References

[ece310998-bib-0001] Allred, B. W. , & Bestelmeyer, B. T. (2021). Improving Landsat predictions of rangeland fractional cover with multitask learning and uncertainty. Methods in ecology and evolution / British Ecological Society, 12, 841–849.

[ece310998-bib-0002] Armbruster, M. J. (1987). Habitat Suitability Index Models: Greater Sandhill Cranes . Available at: https://citeseerx.ist.psu.edu/document?repid=rep1&type=pdf&doi=f996c659e0d239a6b3a6c9313c98ba73b1729f5a

[ece310998-bib-0003] Austin, J. E. , Henry, A. R. , & Ball, I. J. (2007). Sandhill crane abundance and nesting ecology at grays Lake, Idaho. The Journal of Wildlife Management, 71(4), 1067–1079.

[ece310998-bib-0004] Austin, J. E. , Morrison, K. L. , & Harris, J. T. (2018). Cranes and Agriculture: A Global Guide for Sharing the Landscape. International Crane Foundation. Available at: https://www.savingcranes.org/wp‐content/uploads/2018/10/cranes_and_agriculture_web_2018.pdf#page=92

[ece310998-bib-0005] Blevins, S. , Hansen, K. , Paige, G. , & Mac Kinnon, A. (2016). Valuing the non‐agricultural benefits of flood irrigation in the Upper Green River basin . wyagresearch.org. Available at: https://www.wyagresearch.org/research/fdb/2016‐offstation‐valuing‐the‐non‐agricultural‐benefits‐of‐flood‐irrigation.pdf (Accessed: 12 October 2021).

[ece310998-bib-0006] Boggie, M. A. , & Collins, D. P. (2018). Land Use, anthropogenic disturbance, and riverine features drive patterns of habitat selection by a wintering waterbird in a semi‐arid environment. PLoS One, 13(11), e0206222. 30403712 10.1371/journal.pone.0206222 PMC6221299

[ece310998-bib-0007] Breiman, L. (2001). Random forests. Machine Learning, 45(1), 5–32.

[ece310998-bib-0008] Calenge, C. (2011). *Analysis of animal movements in R: the adehabitatLT package* [R]. finzi.psych.upenn.edu. Available at: http://finzi.psych.upenn.edu/library/adehabitatLT/doc/adehabitatLT.pdf

[ece310998-bib-0009] Caro, T. M. , & O'Doherty, G. (1999). ‘On the use of surrogate species in conservation biology. Conservation Biology, 13(4), 805–814.

[ece310998-bib-0010] Collins, D. P. , & Grisham, B. A. (2016). New summer areas and mixing of two greater sandhill crane populations in the Intermountain West. Journal of Fish and Wildlife Management, 7(1), 141–152.

[ece310998-bib-0011] Conring, C. M. (2016). Spatial ecology of the Lower Colorado River Valley population of greater sandhill cranes . Edited by B.A. Grisham. Masters of Sciences. Texas Tech University. Available at: https://ttu‐ir.tdl.org/handle/2346/67141

[ece310998-bib-0012] Cowardin, L. M. , Carter, F. C. , & Golet, E. T. (1979). Classification of wetlands and deepwater habitats of the United States. United States Department of the Interior, Fish and Wildlife Service. Available at: Classification of Wetlands and Deepwater Habitats of the United States.

[ece310998-bib-0013] Culter, D. R. , Edwards, T. C., Jr. , Beard, K. H. , Cutler, A. , Hess, K. T. , Gibson, J. , & Lawler, J. J. (2007). Random Forests for Classification in Ecology. Ecology, 11(88), 2783–2792. 10.1890/07-0539.1 18051647

[ece310998-bib-0014] Donnelly, J. P. , Jensco, K. , Kimball, J. S. , Ketchum, D. , Collins, D. P. , & Naugle, D. E. (2023). Beneficial “inefficiencies” of western ranching: Flood‐irrigated hay production sustains wetland systems by mimicking historic hydrologic processes. *bioRxiv*. 10.1101/2023.12.10.571036

[ece310998-bib-0015] Donnelly, J. P. , & King, S. L. (2020). Climate and human water use diminish wetland networks supporting continental waterbird migration. Global Change Biology, 26(4), 2042–2059. 31967369 10.1111/gcb.15010 PMC7155039

[ece310998-bib-0016] Donnelly, J. P. , & King, S. L. (2021). Migration efficiency sustains connectivity across agroecological networks supporting sandhill crane migration. Ecosphere, 12(6), 1–22.34938591

[ece310998-bib-0017] Donnelly, J. P. , Moore, J. N. , Casazza, M. L. , & Coons, S. P. (2022). Functional Wetland Loss Drives Emerging Risks to Waterbird Migration Networks. Frontiers in Ecology and Evolution, 10, 1–18. 10.3389/fevo.2022.844278

[ece310998-bib-0018] Dozier, A. Q. , & Arabi, M. (2017). Declining agricultural production in rapidly urbanizing semi‐arid regions: policy tradeoffs and sustainability indicators. Environmental Research Letters: ERL [Web site], 12(8), 1–9.36204013

[ece310998-bib-0019] Drewien, R. C. , & Bizeau, E. G. (1974). Status and distribution of greater sandhill cranes in the Rocky Mountains. The Journal of Wildlife Management, 38(4), 720–742.

[ece310998-bib-0020] Drewien, R. C. , Brown, W. M. , & Kendall, W. L. (1995). Recruitment in Rocky Mountain greater sandhill cranes and comparison with other crane populations. The Journal of Wildlife Management, 59(2), 339–356.

[ece310998-bib-0021] Elliott, J. , & Deryng, D. (2014). Constraints and potentials of future irrigation water availability on agricultural production under climate change. Proceedings of the National Academy of Sciences of the United States of America, 111(9), 3239–3244. 24344283 10.1073/pnas.1222474110 PMC3948288

[ece310998-bib-0022] Evans, J. S. , & Murphy, M. A. (2015). Package “rfUtilities”. R package, 1, p. 1.

[ece310998-bib-0023] Fauchald, P. , & Tveraa, T. (2003). Using first‐passage time in the analysis of area‐restricted search and habitat selection. Ecology, 84(2), 282–288.

[ece310998-bib-0024] Foti, R. , & Del Jesus, M. (2012). Hydroperiod regime controls the organization of plant species in wetlands. Proceedings of the National Academy of Sciences of the United States of America, 109(48), 19596–19600. 23150589 10.1073/pnas.1218056109 PMC3511740

[ece310998-bib-0025] Gordon, B. L. , & Paige, G. B. (2020). Field scale quantification indicates potential for variability in return flows from flood irrigation in the high altitude western US. Agricultural Water Management, 232, 1–12.

[ece310998-bib-0026] Gorelick, N. , & Hancher, M. (2017). Google Earth Engine: Planetary‐scale geospatial analysis for everyone. Remote Sensing of Environment, 202, 18–27.

[ece310998-bib-0027] Guisan, A. , & Tingley, R. (2013). Predicting species distributions for conservation decisions. Ecology Letters, 16(12), 1424–1435. 24134332 10.1111/ele.12189 PMC4280402

[ece310998-bib-0028] Hengeveld, R. , & Haeck, J. (1982). The distribution of abundance. I. Measurements. Journal of Biogeography, 9(4), 303–316.

[ece310998-bib-0029] Johnsgard, P. A. (2017). Those of the gray wind: The sandhill cranes, New Edition. U of Nebraska Press.

[ece310998-bib-0030] Johnson, A. R. , & Wiens, J. A. (1992). Animal movements and population dynamics in heterogeneous landscapes. Landscape Ecology, 7(1), 63–75.

[ece310998-bib-0031] Kendy, E. , & Bredehoeft, J. D. (2006). Transient effects of groundwater pumping and surface‐water‐irrigation returns on streamflow. Water Resources Research, 42(8), 1–11. 10.1029/2005wr004792

[ece310998-bib-0032] Kennedy, C. M. , & Oakleaf, J. R. (2019). Managing the middle: A shift in conservation priorities based on the global human modification gradient. Global Change Biology, 25(3), 811–826. 30629311 10.1111/gcb.14549

[ece310998-bib-0201] Ketchum, D., Hoylman, Z. H., Huntington, J., Brinkerhoff, D., & Jencso, K. G. (2023). Irrigation intensification impacts sustainability of streamflow in the Western United States. Communications Earth & Environment, 4(1), 1–8.37325084

[ece310998-bib-0033] Krapu, G. L. , & Brandt, D. A. (2010). Population status and geographic distribution of greater sandhill cranes in the mid‐continent population. In B.K. Hartup (ed.) Proceedings of the Eleventh North American Crane Workshop. North American Crane Workshop, The North American Crane Working Group, p. 12.

[ece310998-bib-0034] Lauenroth, W. K. , Schlaepfer, D. R. , & Bradford, J. B. (2014). Ecohydrology of dry regions: Storage versus pulse soil water dynamics. Ecosystems, 17(8), 1469–1479.

[ece310998-bib-0035] Lavielle, M. (1999). Detection of multiple changes in a sequence of dependent variables. Stochastic Processes and their Applications, 83(1), 79–102.

[ece310998-bib-0036] Lavielle, M. , & Teyssière, G. (2006). Detection of multiple change‐points in multivariate time series. Lithuanian Mathematical Journal, 46(3), 287–306.

[ece310998-bib-0037] Le Corre, M. , Dussault, C. , & Côté, S. D. (2014). Detecting changes in the annual movements of terrestrial migratory species: Using the first‐passage time to document the spring migration of caribou. Movement Ecology, 2(19), 1–11. 27148451 10.1186/s40462-014-0019-0 PMC4855333

[ece310998-bib-0038] Leonetti, C. (2010). Indigenous stewardship methods and NRCS conservation practices . Natural Resources Conservation Service/Native Practices Work Group. Available at: https://efotg.sc.egov.usda.gov/references/public/va/IndigenousStewardship.pdf

[ece310998-bib-0039] Liaw, A. , & Wiener, M. (2002). Classification and regression by randomForest. R News, 2(3), 18–22.

[ece310998-bib-0040] Littlefield, C. D. , Stern, M. A. , & Schlorff, R. W. (1994). Summer distribution, status, and trends of greater sandhill crane populations in Oregon and California. Northwestern Naturalist, 75(1), 1–10.

[ece310998-bib-0041] Liu, C. , Newell, G. , & White, M. (2019). The effect of sample size on the accuracy of species distribution models: Considering both presences and pseudo‐absences or background sites. Ecography, 42(3), 535–548.

[ece310998-bib-0042] Maron, M. , McAlpine, C. A. , & Watson, J. (2015). Climate‐induced resource bottlenecks exacerbate species vulnerability: A review. Diversity, 21(21), 731–743.

[ece310998-bib-0043] McWethy, D. B. , & Austin, J. E. (2009). Nesting ecology of greater sandhill cranes (*Grus canadensis tabida*) in riparian and palustrine wetlands of eastern Idaho. Waterbirds/The Waterbird Society, 32(1), 106–115.

[ece310998-bib-0044] Mi, C. , & Huettmann, F. (2017). Why choose Random Forest to predict rare species distribution with few samples in large undersampled areas? Three Asian crane species models provide supporting evidence. PeerJ, 5, e2849. 28097060 10.7717/peerj.2849 PMC5237372

[ece310998-bib-0045] Mix, K. , Rast, W. , & Lopes, V. L. (2009). Increases in growing degree days in the Alpine Desert of the San Luis Valley, Colorado. Water, Air, and Soil Pollution, 205, 289–304.

[ece310998-bib-0046] Moulton, C. E. , & Carlisle, J. D. (2022). Importance of flood irrigation for foraging colonial waterbirds. The Journal of Wildlife Management, 86(7), 1–15.

[ece310998-bib-0047] Oshiro, T. M. , Perez, P. S. , & Baranauskas, J. A. (2012). How many trees in a random Forest? In P. Perner (Ed.), Machine learning and data Mining in Pattern Recognition. International workshop on machine learning and data Mining in Pattern Recognition (pp. 154–168). Springer Berlin Heidelberg.

[ece310998-bib-0048] Pimm, S. L. , & Alibhai, S. (2015). Emerging Technologies to Conserve Biodiversity. Trends in Ecology & Evolution, 30(11), 685–696. 26437636 10.1016/j.tree.2015.08.008

[ece310998-bib-0049] Pimm, S. L. , & Jenkins, C. N. (2014). The biodiversity of species and their rates of extinction, distribution, and protection. Science, 344(6187), 1246752. 24876501 10.1126/science.1246752

[ece310998-bib-0050] QGIS Development Team . (2020). QGIS . Open Source Geospatial Foundation Project. Available at: http://qgis.osgeo.org

[ece310998-bib-0051] R Core Team . (2019). R: A language and environment for statistical computing [R]. R Foundation for Statistical Computing. Available at: https://www.R‐project.org/

[ece310998-bib-0052] Rawling, M. S. (1992). Distribution and status of greater sandhill cranes in Nevada. In D.A. Wood (ed.) Proceedings of the 5th north American crane workshop. North American Crane Workshop, p. 11.

[ece310998-bib-0053] RStudio Team . (2019). RStudio: Integrated development environment for R. RStudio, Inc. Available at: http://www.rstudio.com/

[ece310998-bib-0054] Sanchez, L. , Edwards, E. C. , & Leonard, B. (2023). Paper water, wet water, and the recognition of indigenous property rights. Journal of the Association of Environmental and Resource Economists [Preprint]. 10(6), 1545–1579. 10.1086/725400

[ece310998-bib-0055] Schaible, G. D. , & Aillery, M. P. (2017). Challenges for US irrigated agriculture in the face of emerging demands and climate change. In J. R. Ziolkowska & J. M. Peterson (Eds.), Competition for water resources (pp. 44–79). Elsevier.

[ece310998-bib-0056] Senner, S. E. , Andres, B. A. , & Gates, H. R. (2016). Pacific Americas shorebird conservation strategy. National Audubon Society. Available at: https://www.fws.gov/migratorybirds/pdf/management/PASCS_final_medres_dec2016.pdf

[ece310998-bib-0057] Simpson, E. H. (1949). Measurement of diversity. Nature, 163, 688.

[ece310998-bib-0058] Snyder, K. A. , & Evers, L. (2019). Effects of Changing Climate on the Hydrological Cycle in Cold Desert Ecosystems of the Great Basin and Columbia Plateau. Rangeland Ecology & Management, 72(1), 1–12.

[ece310998-bib-0059] Sofaer, H. R. , & Jarnevich, C. S. (2019). Development and Delivery of Species Distribution Models to Inform Decision‐Making. Bioscience, 69(7), 544–557.

[ece310998-bib-0060] Swartz, T. M. , & Miller, J. R. (2021). The American Pond Belt: An untold story of conservation challenges and opportunities. Frontiers in Ecology and the Environment, 19(9), 501–509.

[ece310998-bib-0061] Thornton, M. M. , Shrestha, R. , Wei, Y. , Thornton, P. E. , Kao, S.‐C. , & Wilson, B. E. (2022). Daymet: daily surface weather data on a 1‐km grid for North America, Version 4. ORNL DAAC. daac.ornl.gov. Available at: https://daac.ornl.gov/DAYMET/guides/Daymet_Daily_V4R1.html

[ece310998-bib-0062] Thorpe, P. P. , Donnelly, J. P. , & Collins, D. P. (2022). September 2022 Survey of the Rocky Mountain Population of Greater Sandhill Cranes. US Fish and Wildlife Service, Division of Migratory Birds. Available at: https://www.fws.gov/sites/default/files/documents/rocky‐mountain‐population‐greater‐sandhill‐crane‐survey‐2022.pdf

[ece310998-bib-0063] Thorvaldson, J. , & Pritchett, J. G. (2006). Economic impact analysis of reduced irrigated acreage in four river basins in Colorado. Colorado Water Resources Research Institute. Available at: https://watercenter.colostate.edu/wp‐content/uploads/sites/33/2020/03/CR207.pdf

[ece310998-bib-0064] Tiner, R. W. (2003). Estimated extent of geographically isolated wetlands in selected areas of the United States. Wetlands, 23(3), 636–652.

[ece310998-bib-0065] VanDerWal, J. , & Shoo, L. P. (2009). Selecting pseudo‐absence data for presence‐only distribution modeling: How far should you stray from what you know? Ecological Modelling, 220(4), 589–594.

[ece310998-bib-0066] Welsh, L. W. , & Endter‐Wada, J. (2017). Piping water from rural counties to fuel growth in Las Vegas, Nevada: Water transfer risks in the arid USA west. Water Alternatives, 10(2), 420.

[ece310998-bib-0067] Wickham, H. , & Averick, M. (2019). Welcome to the tidyverse. Journal of Open Source Software, 4(43), 1686.

[ece310998-bib-0068] Wiens, J. A. , & Stralberg, D. (2009). Niches, models, and climate change: Assessing the assumptions and uncertainties. Proceedings of the National Academy of Sciences of the United States of America, 106(Suppl 2), 19729–19736. 19822750 10.1073/pnas.0901639106 PMC2780938

[ece310998-bib-0069] Wiken, E. , Jiménez Nava, F. , & Griffith, G. (2011). North American terrestrial ecoregions—Level III. Commission for Environmental Cooperation. Available at: https://gaftp.epa.gov/EPADataCommons/ORD/Ecoregions/pubs/NA_TerrestrialEcoregionsLevel3_Final‐2june11_CEC.pdf

[ece310998-bib-0070] Zhuang, W. (2016). Eco‐environmental impact of inter‐basin water transfer projects: A review. Environmental Science and Pollution Research, 23(13), 12867–12879. 27178293 10.1007/s11356-016-6854-3

