# Supplementary material for: Flood‐irrigated agriculture mediates climate‐induced wetland scarcity for summering sandhill cranes in western North America
Source: Ecol Evol. 2024 Mar 5;14(3):e10998. doi: 10.1002/ece3.10998 (PMC10915483; doi:10.1002/ece3.10998)
Supplement: Supplementary file 2 — Appendix S2. [file ECE3-14-e10998-s001.pdf]

Appendix B - Sandhill crane wetland trend and land ownership summaries

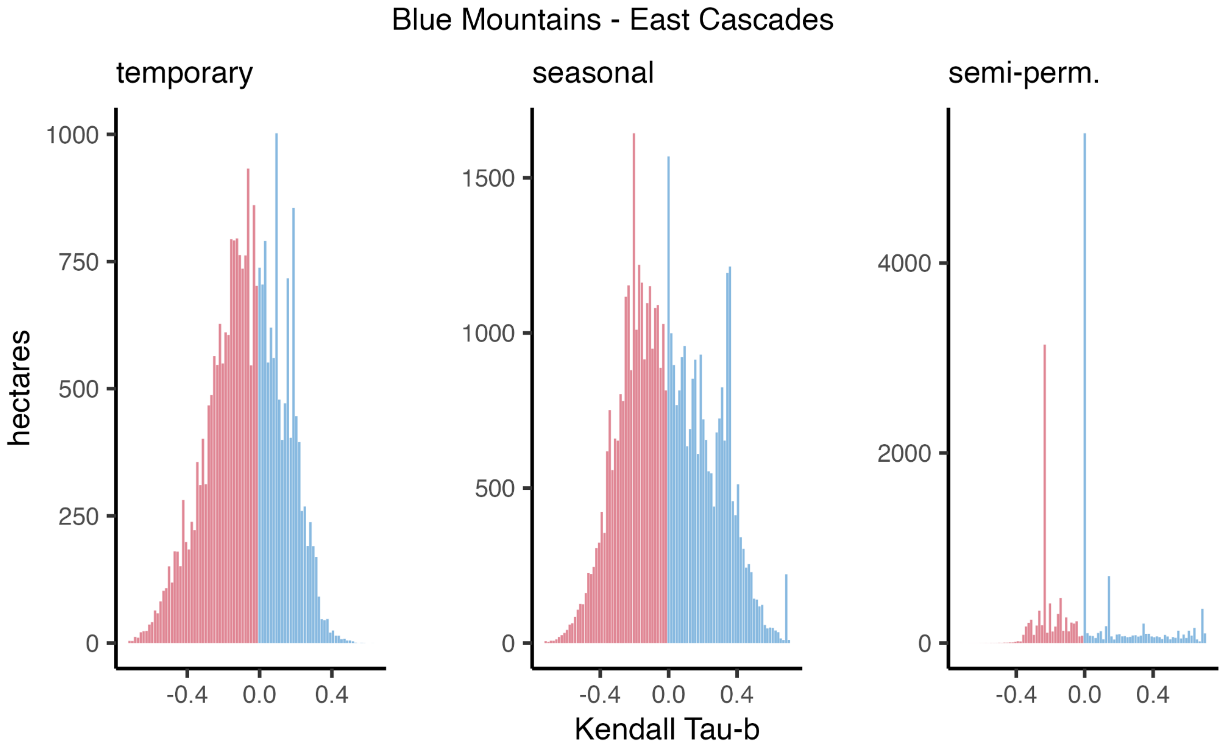

Figure B2. Blue Mountains - East Cascades Kendall's Tau-b rank correlation value distributions by wetland hydroperiod class. Summaries represent all wetlands within sandhill crane core areas. Negative values indicate wetland drying associated with reduced duration or frequency of flooding.

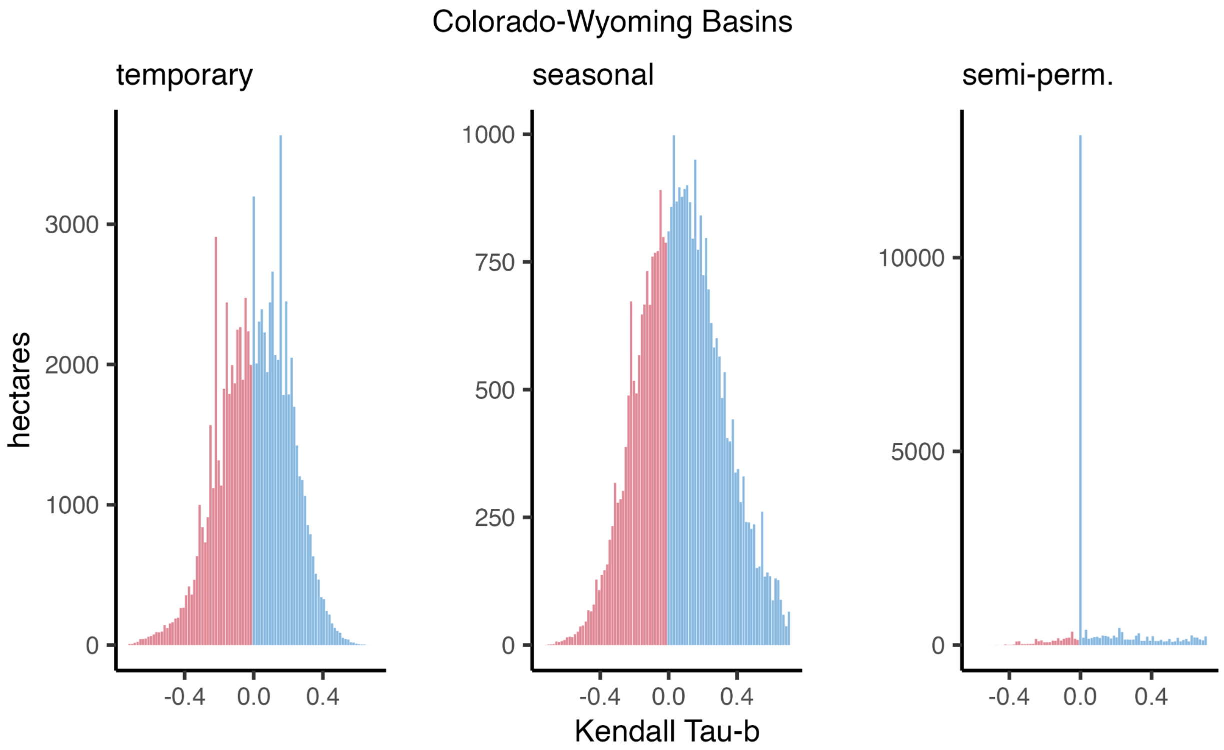

Figure B3. Wyoming - Colorado Bains Kendall's Tau-b rank correlation value distributions by wetland hydroperiod class. Summaries represent all wetlands within sandhill crane core areas. Negative values indicate wetland drying associated with reduced duration or frequency of flooding.

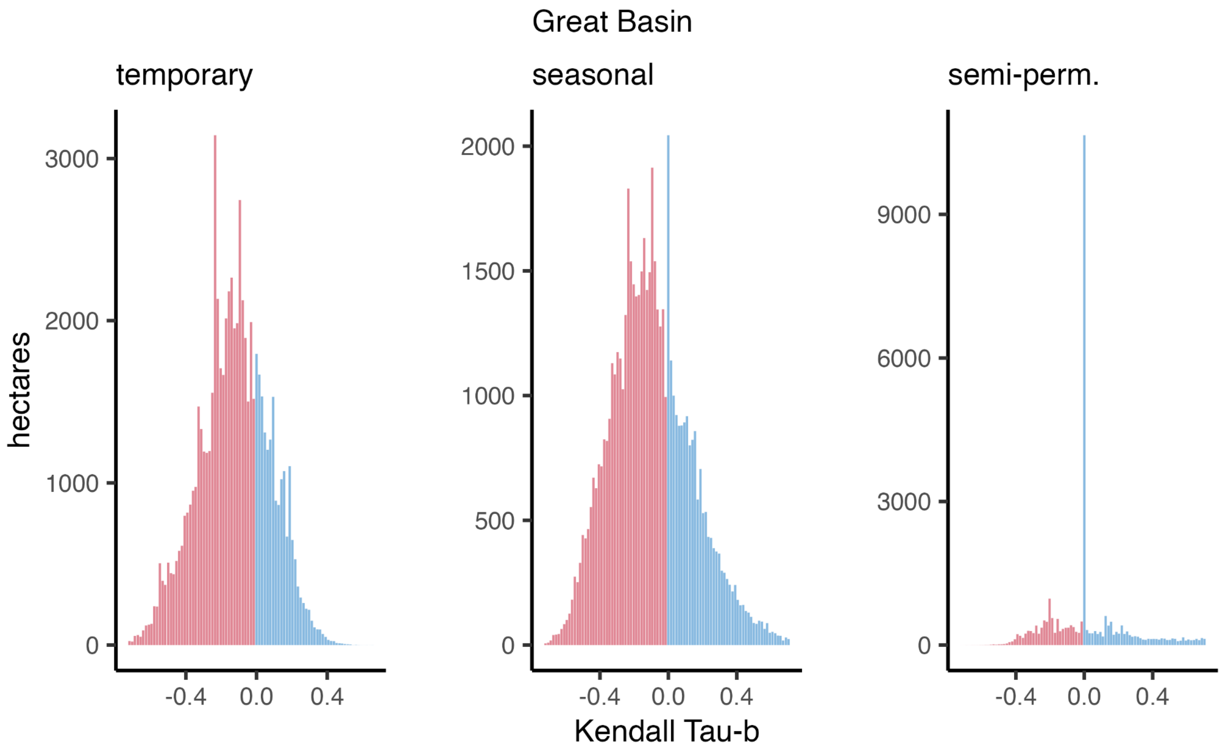

Figure B4. Great Basin Kendall's Tau-b rank correlation value distributions by wetland hydroperiod class. Summaries represent all wetlands within sandhill crane core areas. Negative values indicate wetland drying associated with reduced duration or frequency of flooding.

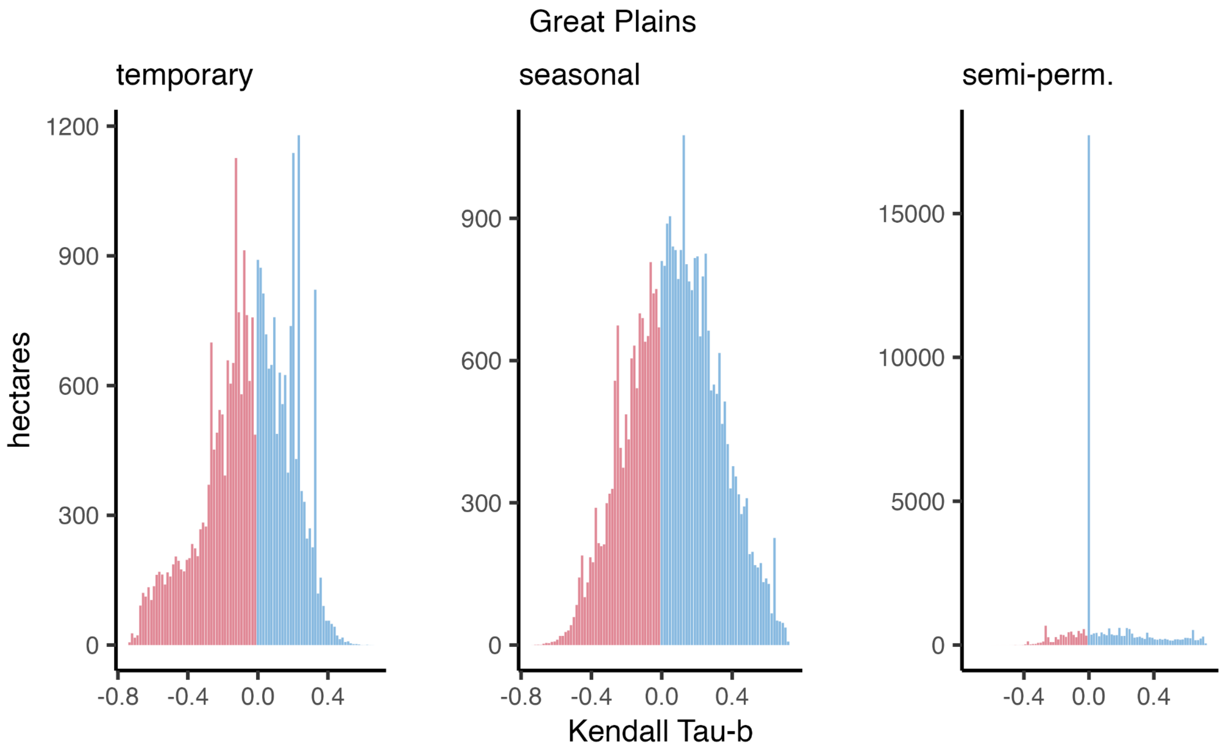

Figure B5. Great Plains Kendall's Tau-b rank correlation value distributions by wetland hydroperiod class. Summaries represent all wetlands within sandhill crane core areas. Negative values indicate wetland drying associated with reduced duration or frequency of flooding.

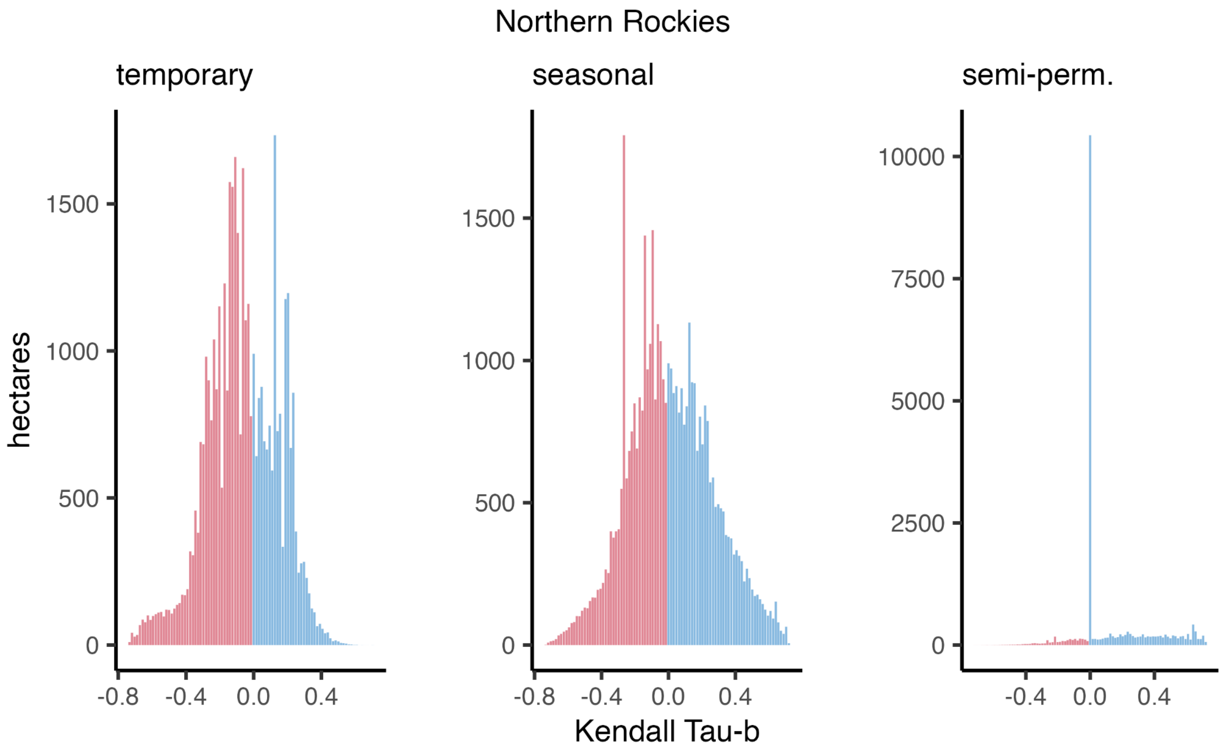

Figure B6. Northern Rockies Kendall's Tau-b rank correlation value distributions by wetland hydroperiod class. Summaries represent all wetlands within sandhill crane core areas. Negative values indicate wetland drying associated with reduced duration or frequency of flooding.

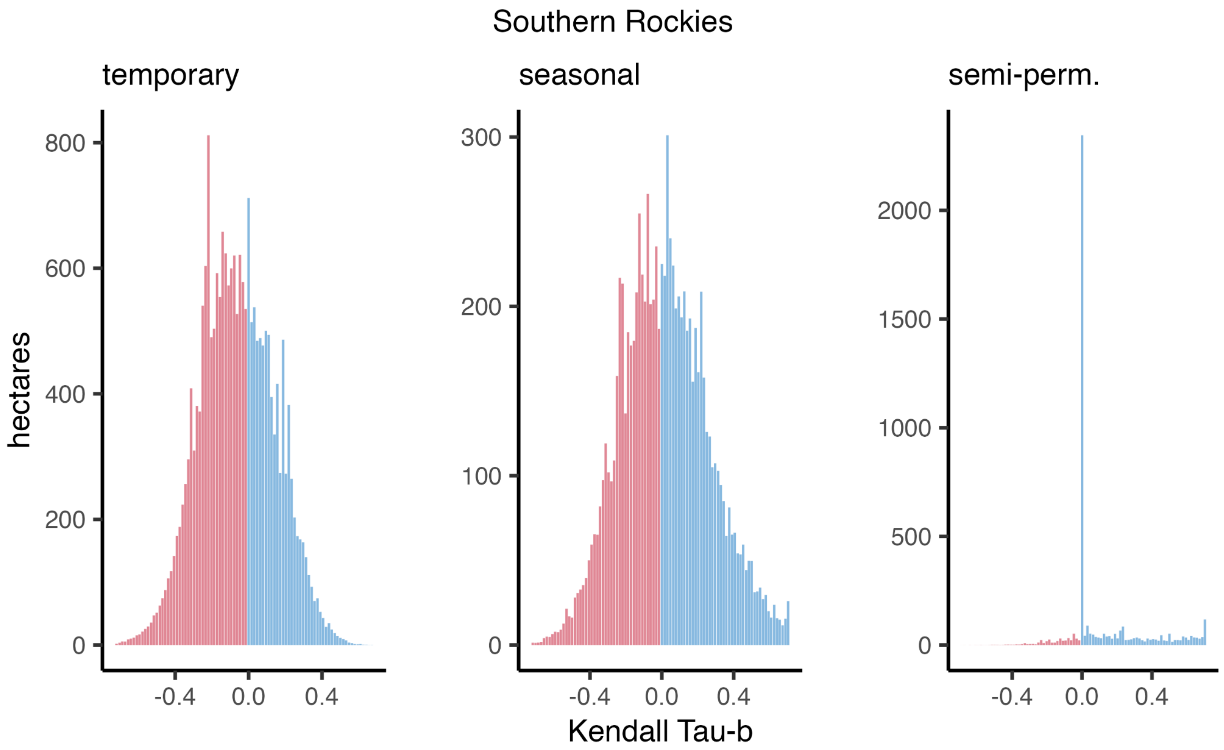

Figure B7. Southern Rockies Kendall's Tau-b rank correlation value distributions by wetland hydroperiod class. Summaries represent all wetlands within sandhill crane core areas. Negative values indicate wetland drying associated with reduced duration or frequency of flooding.

Table B1. Sandhill crane core summering area summarized by public land management agency. Percentages represent proportional abundance within the overall core area. Other public lands class represents an amalgamation of over 15 agencies and municipalities that individually account for $\leq$ 0.3% of core summering areas.

Public land management agency	hectares	percentage
U.S. Forest Service	57,439	4.5%
Bureau of Land Management	34,382	2.7%
U.S. Fish and Wildlife Service	31,963	2.5%
State Wildlife Agencies	18,704	1.5%
National Park Service	10,612	0.8%
U.S. Bureau of Reclamation	5,916	0.5%
Other public lands	43,268	3.4%

Table B2. Temporary wetlands summarized by public land management agency. Percentages represent proportional abundance within the overall sandhill crane core summering area. Other public lands represent an amalgamation of minor occurrences from over 15 agencies and municipalities.

Public land management agency	hectares	percentage
U.S. Fish and Wildlife Service	22,969	3.1%
U.S. Forest Service	16,867	2.3%
State Wildlife Agencies	13,074	1.8%
Bureau of Land Management	10,580	1.4%
National Park Service	3,838	0.5%
Other public lands	21,260	2.9%

Table B3. Seasonal wetlands summarized by public land management agency. Percentages represent proportional abundance within the overall sandhill crane core summering area. Other public lands represent an amalgamation of minor occurrences from over 15 agencies and municipalities.

Public land management agency	hectares	percentage
U.S. Fish and Wildlife Service	60,676	10.7%
U.S. Forest Service	16,867	5.8%
State Wildlife Agencies	29,244	5.2%
Bureau of Land Management	20,381	3.6%
National Park Service	7,525	1.4%
Other public lands	21,911	3.9%

Table B4. Semi-permanent wetlands summarized by public land management agency. Percentages represent proportional abundance within the overall sandhill crane core summering area. Other public lands represent an amalgamation of minor occurrences from over 15 agencies and municipalities.

Public land management agency	hectares	percentage
U.S. Fish and Wildlife Service	42,673	12.9%
U.S. Forest Service	8,269	2.5%
State Wildlife Agencies	29,365	8.9%
Bureau of Land Management	15,576	4.7%
National Park Service	2,037	0.6%
Other public lands	8,387	3.6%
